# Recording Neural Activity Based on Surface Plasmon Resonance by Optical Fibers-A Computational Analysis

**DOI:** 10.3389/fncom.2018.00061

**Published:** 2018-08-03

**Authors:** Mitra Abedini, Tahereh Tekieh, Pezhman Sasanpour

**Affiliations:** ^1^Department of Medical Physics and Biomedical Engineering, School of Medicine, Shahid Beheshti University of Medical Sciences, Tehran, Iran; ^2^Complex System Group, Department of Physics, Sydney University, Sydney, NSW, Australia; ^3^School of Nanoscience, Institute for Research in Fundamental Sciences (IPM), Tehran, Iran

**Keywords:** neural activity recording, action potential, surface plasmon resonance, fiber optics, COMSOL, optical recording, optogenetics

## Abstract

An all optical, non-destructive method for monitoring neural activity has been proposed and its performance in detection has been analyzed computationally. The proposed method is based on excitation of Surface Plasmon Resonance (SPR) through the structure of optical fibers. The sensor structure consists of a multimode optical fiber where, the cladding of fiber has been removed and thin film of gold structure has been deposited on the surface. Impinging the laser light with appropriate wavelength inside the fiber and based on the total internal reflection, the evanescent wave will excite surface plasmons in the gold thin film. The absorption of light by surface plasmons in the gold structure is severely dependent on the dielectric properties at its vicinity. The electrical activity of neural cells (action potential) can modulate the dielectric properties at its vicinity and hence can modify the absorption of light inside the optical fiber. We have computationally analyzed the performance of the proposed sensor with different available geometries using Finite Element Method (FEM). In this regard, we have shown that the optical response of proposed sensor will track the action potential of the neuron at its vicinity. Based on different geometrical structure, the sensor has absorption in different regions of visible spectrum.

## Introduction

Neurons as the main building blocks of central (brain and spinal cord) and peripheral nervous system are well known. Processing and communication of data in various parts of nervous system are performed basically through the propagating variation of transmembrane electric potential of neurons, called action potentials. The electrophysiological function of neurons is the core of memory, cognition, movement, and autonomic functions. Detection, monitoring and recording the electrophysiological activity of neuron(s) are the most important subjects of neurophysiology. The basic therapeutic studies on the various types of neurological disorders including Alzheimer, Parkinson, Multiple Sclerosis (MS), and traumatic brain injuries requires the real time monitoring of the neural activity (Kempuraj et al., 2017). The fundamental studies on the different treatments for the neuron based disorders such as pharmacological treatments, excitation with electric/magnetic fields and stem cell therapy are dependent to the devices in which the electrical activity of the neurons are monitored in real time (Eissa et al., 2018; Tekieh et al., 2018; Zhong et al., 2018). In addition, the advanced studies on the performance of the brain in cognitive neuroscience applications are from demanding areas of monitoring the electrical signaling and activity in different regions of brain (Chen et al., 2016; Hindriks et al., 2016; Sokolov et al., 2016; Deadwyler et al., 2017). The spatial resolution, sensitivity, signal to noise ratio and biocompatibility are the key constraints of different methods in this regard.

Due to the importance of neuron's electrophysiological activity, there have been different proposed techniques of recording the electrical activities and stimulating the neurons. Electrical recording of neural activity using multi electrode structures with different geometries of sharp needles, planar electrodes, and flexible substrates are performed with microelectronics fabricated structures(Gunasekera et al., 2015; Maccione et al., 2015; Chen et al., 2017). Based on the intra/extra cellular recording, geometry of the electrode is an important parameter to consider. Wires and micropipette structures are intruding the cell structure for intracellular recordings, while the planar structures are designed for extracellular recordings (Chen et al., 2017). From material point of view, there have been different types of material (e.g., silicon, platinum, tungsten and gold) applied for fabrication of electrodes. To select the electrode's material and geometry the electrical impedance, signal to noise ratio and biocompatibility should be considered. With the advent of nanostructures and based on their motivating electrical and mechanical properties, there have been different approaches based on the application of nanostructures in extra/intra cellular recordings. Different 1D/2D nanostructures including carbon nanotubes (CNT) and graphene have been applied in fabrication of electrodes (Hanein and Bareket-Keren, 2013; Fabbro et al., 2016; Park et al., 2016). Application of nanostructured electrodes will have the fundamental benefits including higher surface area, electrical conductivity and enhanced adhesion of cell to the substrate (Marchesan et al., 2017; Scaini and Ballerini, 2018). It has been shown that, carbon nanotubes have the potential to mimic the intracellular electrodes. Considering neural prosthesis, CNT structures have also shown the great potential for neural cell cultivation on their surface (Cellot et al., 2011; Giugliano et al., 2012). Graphene based structures have been applied as substrates for stimulation, considering their good adhesion, biocompatibility and great charge injection capacity (Park et al., 2018). Although very applicable, but there are some limitations in the electrical stimulation and recording of action potential. Artifacts in electrical stimulation, electromagnetic interference, and existing tissue damages, have directed the trend to the non-electrical stimulation and recording. There have been various techniques proposed based on exploiting magnetic field (Barry et al., 2016), acoustic waves (Neely et al., 2018) and optics (Kralj et al., 2011; Lu et al., 2018). Different frequency regions and intensity of above mentioned fields will control the penetration depth consequently.

Optical methods of recording neural activity and stimulation of neurons are performed using different techniques. Infrared excitation (Wells et al., 2005; Shapiro et al., 2012), voltage sensitive fluorescent tags (Kulkarni and Miller, 2017; Liu et al., 2017; Nixima et al., 2017), genetically encoding opsins (Chow et al., 2012), and surface plasmon resonance techniques (Kim et al., 2008) are from well-known methods in this regard. Fluorescence-based techniques using voltage sensitive dyes suffer from different problems and difficulties including bleaching, toxicity, labeling procedures and the instrumentation requirements.

Surface plasmon resonance, the collective resonant oscillation of electrons at the interface of metal and dielectric in response to excitation light, has been exploited in different sensing applications (Csáki et al., 2018; Hinman et al., 2018; Lertvachirapaiboon et al., 2018). The maximum absorption of energy from incident light will be occurred in a specific wavelength which is strictly dependent to the dielectric properties of interface. From application point of view, the absorption properties in SPR sensors could provide the base for monitoring the variation in the refractive index of the interface. The detection could be done based on different methods such as wavelength, intensity and angular techniques. As the electric potential can modify the dielectric properties of the ionic liquid, the SPR has been applied along with electrochemical impedance spectroscopy (EIS) techniques (Polonschii et al., 2014). Depending on its fast, label free and high sensitivity response, SPR has been used to monitor neural activity (Kim et al., 2008). The prism based technique has been applied to monitor the activity of nerves *in vitro*. Using gold nanoparticles, the neural activity has been monitored *in vitro* (Zhang et al., 2009). Optical fibers are great candidates for transferring light inside the body. The main mechanism of light trapping inside the fiber optics is total internal reflection, the same phenomena applied in prism based technique for excitation of surface plasmon modes in the metallic thin film. Based on their size and geometry, the optical fibers can be easily applied for *in vivo* applications. The fiber based SPR sensors have been applied for monitoring different biological samples including bacteria, DNA and specific gravity of urine (O'Keeffe et al., 2015; Liang et al., 2016; Zuppolini et al., 2017). The technique has also been applied for monitoring the neural activity *in vivo* (Kim et al., 2012).

In this paper, a method has been proposed for detection of the neural activity using optical fibers. We have also studied the effect of different available geometrical structures for fabrication of fiber based SPR sensor on the performance of operation. In this regard, based on computational modeling, the performance of three different structures has been evaluated.

## Materials and methods

### Proposed method structure

**Figure 1:**
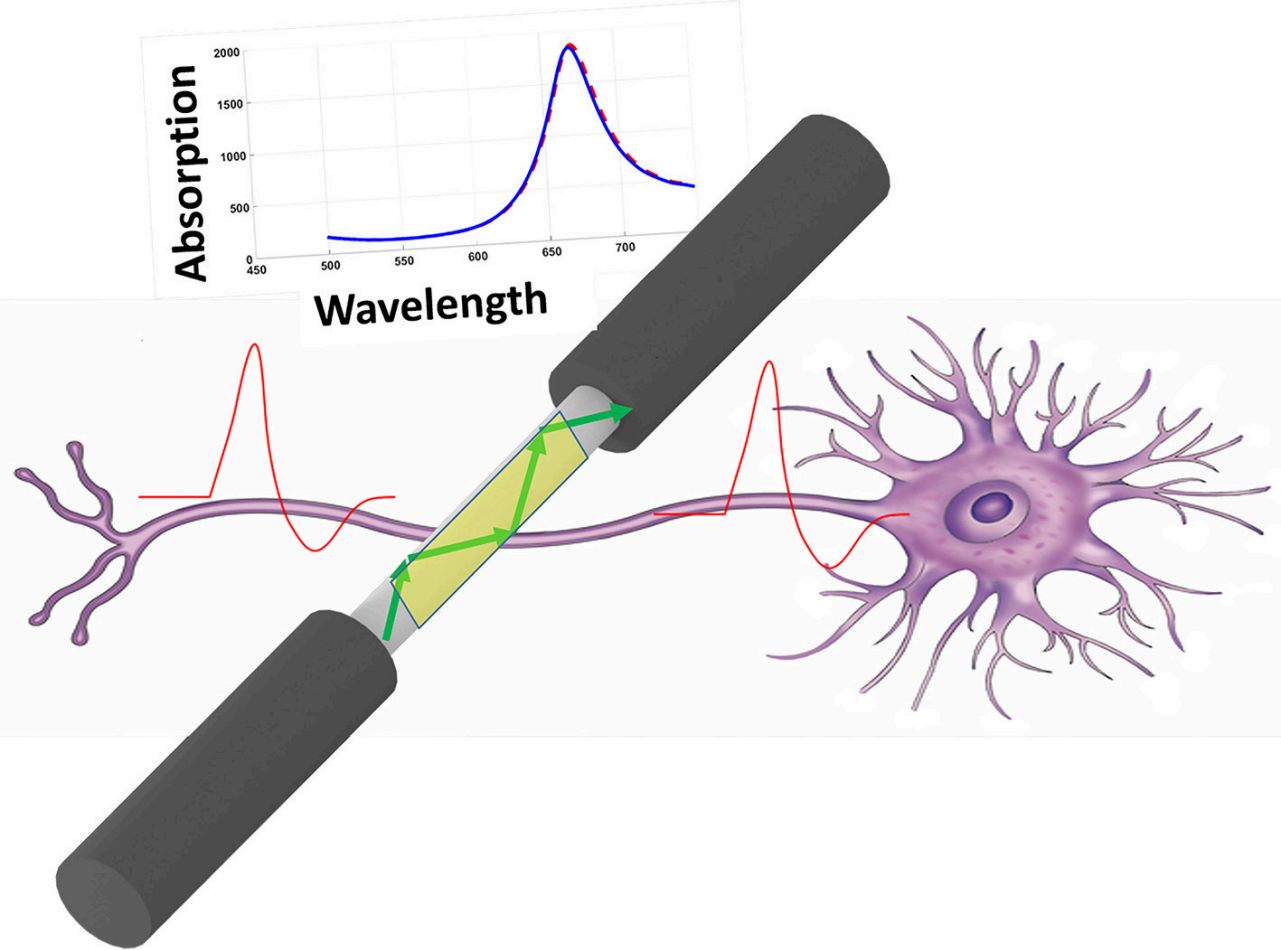
Schematic of recording method based on SPR inside the fiber.

**Figure 2:**
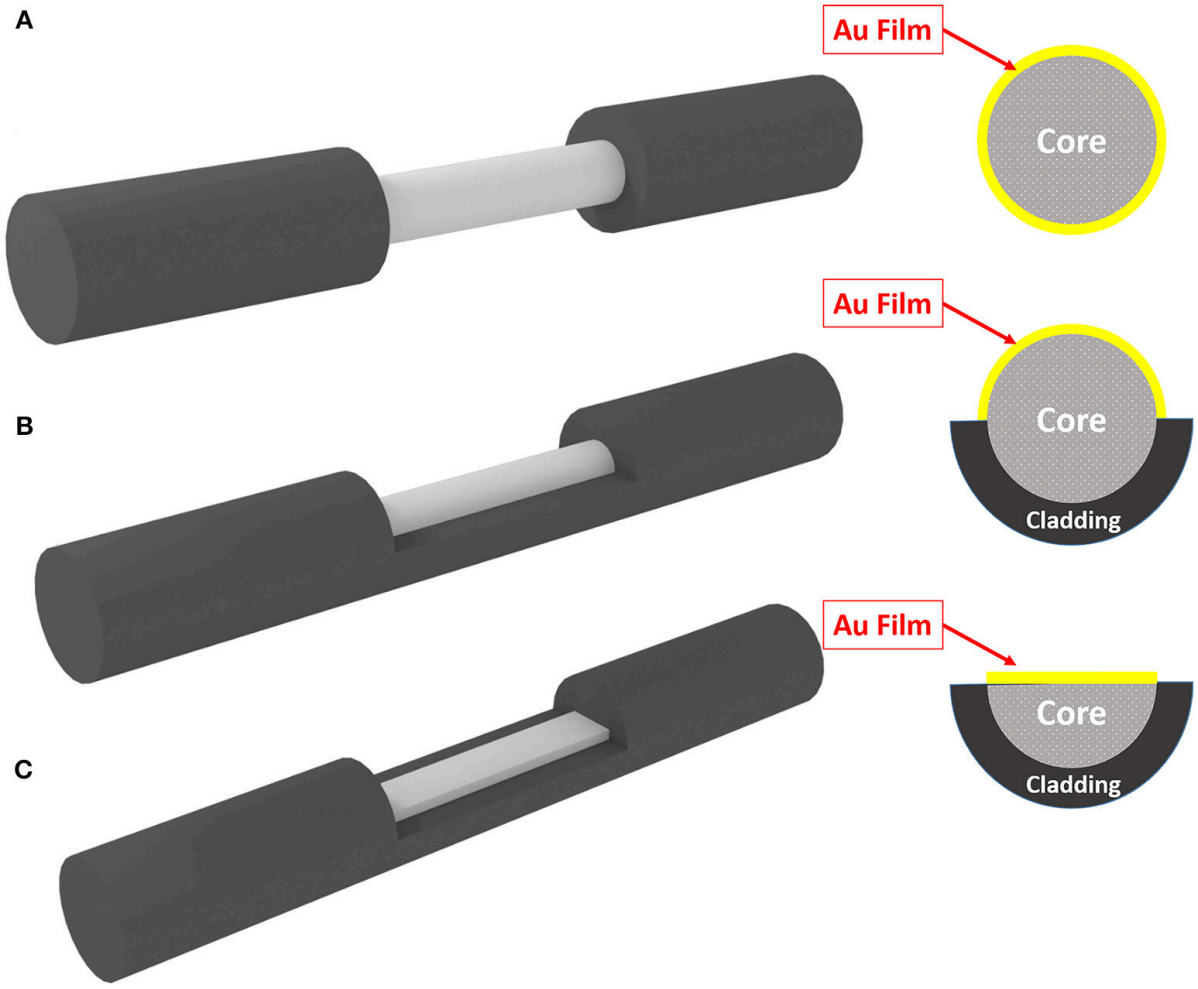
Three different geometrical structure of fiber for SPR. **(A)** Fully-removed cladding. **(B)** Half-removed cladding. **(C)** Half-removed core.

The electrical activity of neuron (action potential) will modify the free electron density inside the gold structure and finally will modify the dielectric constant of the gold structure. Figure 1 shows the schematic of method and its operation. As depicted simply in Figure 1, the variation of dielectric properties of gold will be resulted in the absorption of surface plasmons which are excited in gold through fiber structure (total internal reflection). The variation in absorption is monitored from the output of fiber accordingly. In this study, we have considered three different fiber structures to analyze. In the first case the cladding of fiber is totally removed and replaced with deposited thin film on the surface of core, shown in Figure 2A. As for the second structure, half of the cladding is removed and thin film of gold is deposited instead (Figure 2B). In the third case, half of both cladding and core are removed and the gold layer is deposited (Figure 2C).

### Computational modeling

The absorption spectrums of the optical fibers have been evaluated based on solving the wave equation inside the fiber structure computationally. The cross section of fiber structure has been considered for 2D analysis. The Finite Element Method (FEM) is used through COMSOL Multiphysics environment to solve Equation (1).


(1) $$\begin{array}{l} \nabla \times \nabla \times \vec{E}-k_{0}^{2}\varepsilon_{0}\varepsilon_{r}\vec{E}=0 \end{array}$$

Where E demonstrates the electric field component of light. k_0_ shows the wave number in vacuum, and ε_0_ and ε_*r*_ represent vacuum permittivity and material's relative permittivity respectively.

For each wavelength the wave equation is solved for the structure to derive the available modes and their propagation constants.

### Effect of neural activity on the permittivity of gold

For the metallic structures, generally the dispersion behavior of permittivity is demonstrated by Drude-Lorentz model, Equation (2).


(2) $$\begin{array}{l} \varepsilon_{\text{r}}\left(\omega \right)=\varepsilon_{\infty }-\frac{\omega_{\text{D}}^{\text{2}}}{\omega^{\text{2}}+\text{i}\gamma_{\text{D}}\omega }-\frac{\omega_{\text{L}}^{\text{2}}\Delta \varepsilon }{\omega^{\text{2}}+\text{i}\gamma_{\text{L}}\omega -\omega_{\text{L}}^{\text{2}}} \end{array}$$

Where ε_∞_ shows the permittivity at high frequencies, γ_*D*_ is damping factor and ω_*D*_ is plasma frequency. ω_*D*_ and γ_*D*_ demonstrate the strength and spectral width of Lorentz oscillator and Δε shows the weighting factor.

Considering the neural activity, the resulted electric field will modify the plasma frequency of gold based on Equation (3) (Huang et al., 2013).


(3) $$\begin{array}{l} \omega_{\text{D}\text{f}}=\sqrt{\text{1}+\frac{\Delta \text{N}}{\text{N}}}\omega_{\text{D}} \end{array}$$

Where N represents the free electron density and ΔN shows the excess charge residing on gold. In our analysis, the electric field distribution around the metallic thin film has been considered uniform.

## Results and discussion

**Figure 3:**
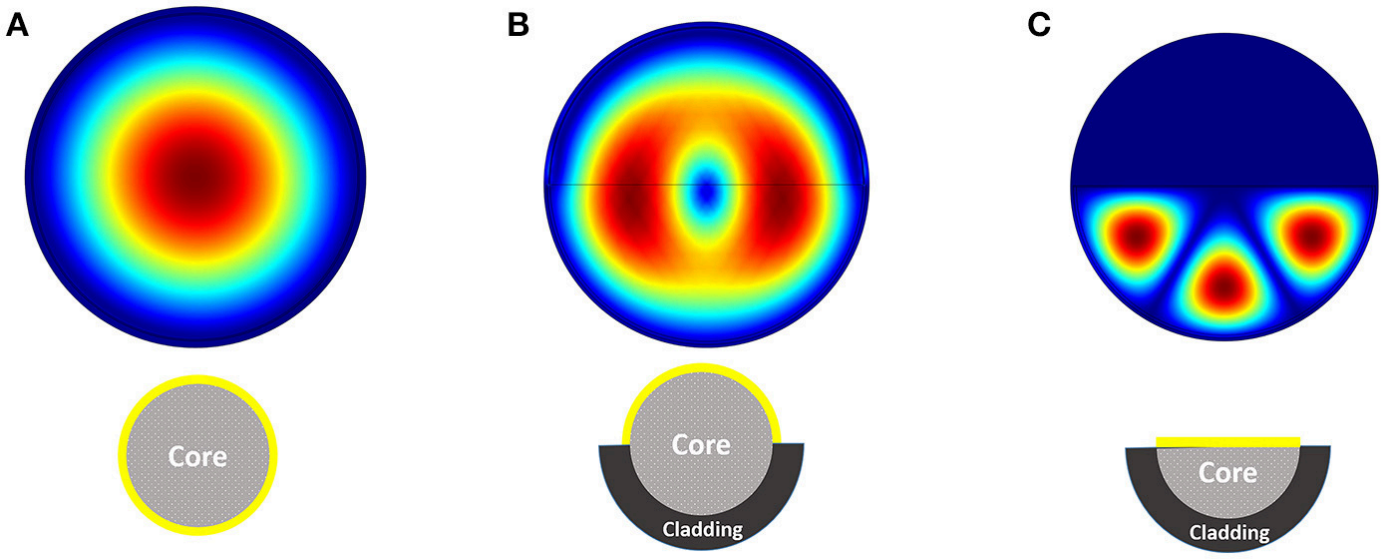
Electric field distribution inside the fiber considering three different structures **(A–C)**.

We have performed the analysis for the structure of proposed sensor with a multi-mode fiber with radius of 5 μm. Fifty nanometer of gold layer has been considered as active element on the surface of core (replaced with cladding). In order to mimic the real biological environment, 1X PBS buffer solution has been considered as the surrounding medium of the fiber structure. Based on different available propagation modes, there will be different electric field distribution inside the optical fiber structure. Figure 3 shows the electric field distribution inside three different structures of Figure 2. The distribution of electric field inside the structure is dictated based on boundary condition, excitation wavelength and the material property. It can be easily seen that the modes inside the fiber with half core and half cladding is arranged to confine the field inside half of the fiber.

**Figure 4:**
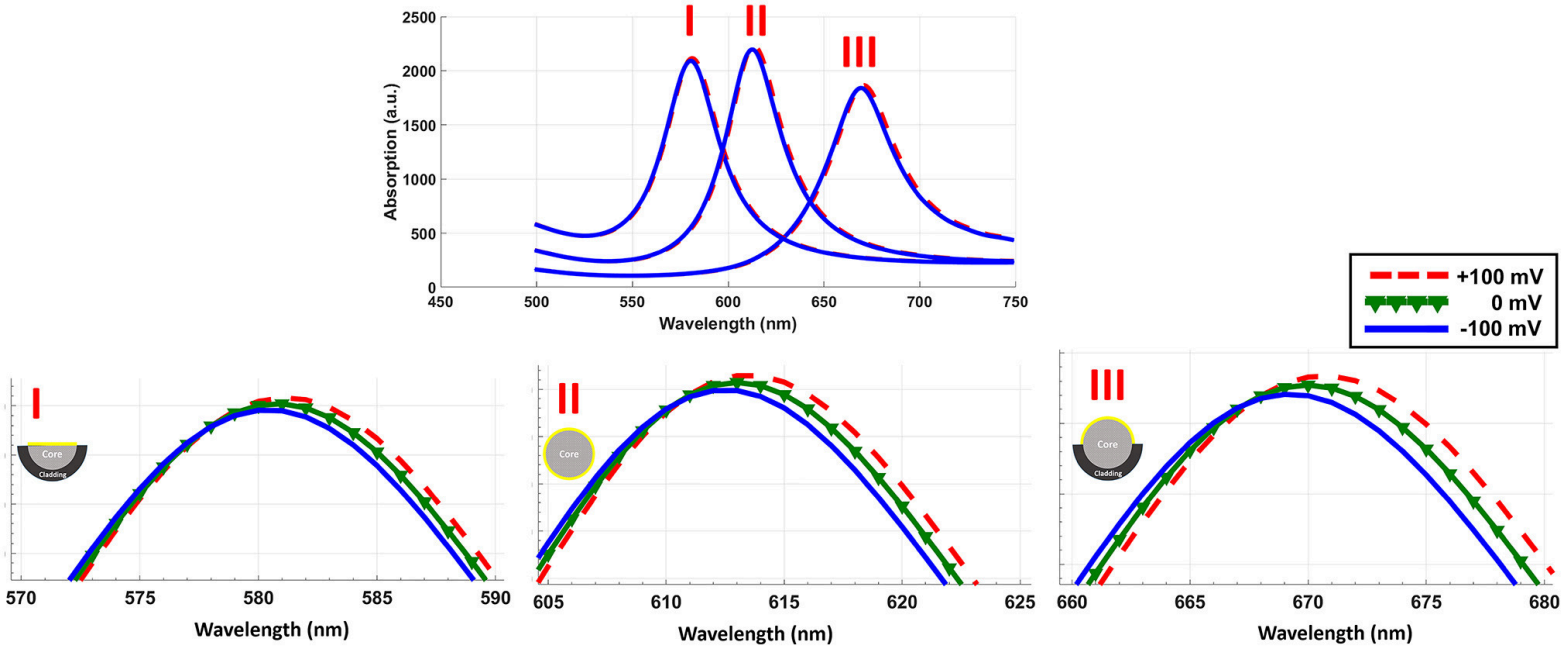
Absorption spectrum of three different geometrical structures in response to electric potential. The inset shows the magnified peaks.

**Figure 5:**
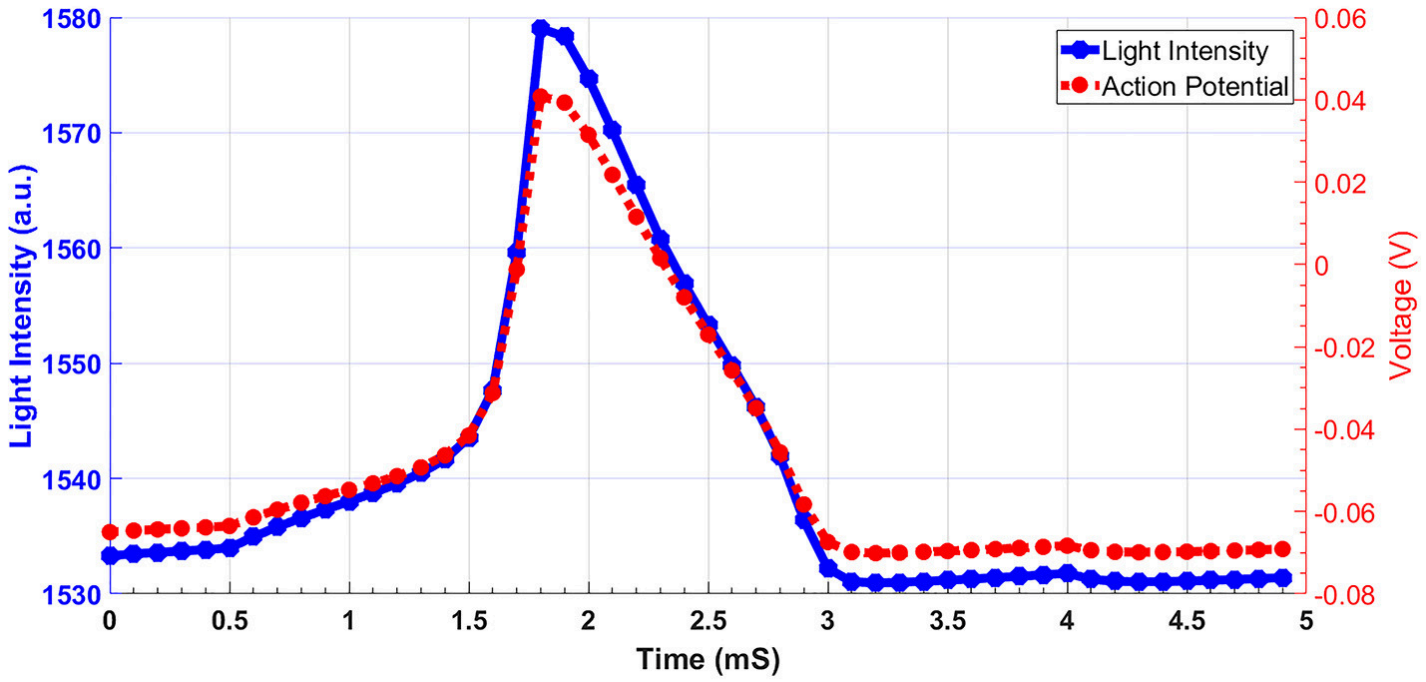
Light intensity of fiber sensor in response to action potential for half-removed cladding. The excitation wavelength is 680 nm.

Considering the range of voltages in action potential, we have calculated the absorption spectrum of structure for −100 and +100 mV. Figure 4 shows the absorption spectrum for three different fiber structures in −100, 0, and +100 mV. As it can be seen, different geometries of fibers have their absorption peak in different wavelengths. For each case the magnified insets show the maximum absorption peak. The shift in peak wavelengths in response to applied voltage (from −100 to +100 mV) was1 nm for fully-removed cladding and half-removed core designs, while a shift of 2 nm was calculated for the half-removed cladding design. In order to check the capability of the proposed structure to track the neural response (action potential pattern), based on the spectral response of the sensor, we have calculated the optical intensity for different voltage ranges in action potential. In this regard, Figure 5 shows the optical response for half-removed cladding structures. As shown, the variation of intensity, tracks the pattern of action potential with appropriate level. Considering optical window for biomedical applications, it seems that the structure with absorption peak around 670 nm could be appropriate in this regard. For intensity-based measurement, the proper wavelength for excitation can be selected based on the absorption spectrum of the structures accordingly. In terms of fabrication, the deposition of thin film of gold on the surface usually is performed using sputtering or thermal evaporation technique. In this regard having a uniformly deposition of the entire perimeter of the circular structure of optical fiber is necessary and needs extra modification in the deposition technique. Meanwhile the half removed cladding structure can be deposited like a planar surface. The half-removed cladding structure shows slightly higher wavelength sensitivity to the voltage, also from the fabrication point of view, the fabrication of this structure is not only more desirable but more reliable.

## Conclusion

The performance of an all optical proposed method for recording neural activity has been evaluated computationally. The analysis was based on solving the wave equation in the fiber and metallic thin film structure by finite element method. The results indicated that the proposed method with different available geometries of fibers are sensitive to the neural activity in their vicinity. Considering wavelength and method of fabrication, the optimum geometry of the structure is for the optical fibers where half cladding has been removed and thin film of gold layer has been deposited therefore. The proposed structure is sensitive, biocompatible, non-destructive, and secure from electromagnetic interference and its performance for monitoring action potential is considerable.

## Author contributions

PS proposed the idea and with the contribution of TT performed the initial simulations. PS and MA performed the finite element simulations. PS analyzed the results and wrote the manuscript. TT and MA assisted in analysis of data, discussions and writing the manuscript. All authors read and edited the manuscript.

### Conflict of interest statement

The authors declare that the research was conducted in the absence of any commercial or financial relationships that could be construed as a potential conflict of interest. The handling Editor declared a shared affiliation, though no other collaboration, with one of the authors PS.
