# Corrigendum: Recording Neural Activity Based on Surface Plasmon Resonance by Optical Fibers-A Computational Analysis

**DOI:** 10.3389/fncom.2018.00087

**Published:** 2018-10-16

**Authors:** Mitra Abedini, Tahereh Tekieh, Pezhman Sasanpour

**Affiliations:** ^1^Department of Medical Physics and Biomedical Engineering, School of Medicine, Shahid Beheshti University of Medical Sciences, Tehran, Iran; ^2^Complex System Group, Department of Physics, Sydney University, Sydney, NSW, Australia; ^3^School of Nanoscience, Institute for Research in Fundamental Sciences (IPM), Tehran, Iran

**Keywords:** neural activity recording, action potential, surface plasmon resonance, fiber optics, COMSOL, optical recording, optogenetics

In the published article, there was an error in affiliation 1. Instead of Department of Medical Physics and Biomedical Engineering, School of Medicine, Shahid Beheshti Medical University, Tehran, Iran, it should be Department of Medical Physics and Biomedical Engineering, School of Medicine, Shahid Beheshti University of Medical Sciences, Tehran, Iran. The authors apologize for this error and state that this does not change the scientific conclusions of the article in any way.

The original article has been updated.

## Conflict of interest statement

The authors declare that the research was conducted in the absence of any commercial or financial relationships that could be construed as a potential conflict of interest.

